# Heart dysfunction in patients with acute ischemic stroke or TIA does not predict all-cause mortality at long-term follow-up

**DOI:** 10.1186/1471-2377-13-122

**Published:** 2013-09-23

**Authors:** Alexandra Holmström, Michael LX Fu, Clara Hjalmarsson, Lena Bokemark, Björn Andersson

**Affiliations:** 1 Department of Clinical and Molecular Medicine, Institute of Medicine, Sahlgrenska Academy, University of Gothenburg, Gothenburg, Sweden; 2 Department of Internal Medicine, Institute of Medicine, Sahlgrenska Academy, University of Gothenburg, Gothenburg, Sweden; 3 Department of Internal Medicine, Sahlgrenska University Hospital/Sahlgrenska, Blå Stråket 5, 413 45 Göteborg, Sweden

**Keywords:** Echocardiography, Heart failure, Mortality, Stroke, TIA

## Abstract

**Background:**

Despite heart failure being a substantial risk factor for stroke, few studies have evaluated the predictive value of heart dysfunction for all-cause mortality in patients with acute ischemic stroke, in particular in the elderly. The aim of this study was to investigate whether impaired heart function in elderly patients can predict all-cause mortality after acute ischemic stroke or transient ischemic attack (TIA).

**Methods:**

A prospective long-term follow-up analysis was performed on a hospital cohort consisting of n = 132 patients with mean age 73 ± 9 years, presenting with acute ischemic stroke or transient ischemic attack, without atrial fibrillation. All patients were examined by echocardiography during the hospital stay. Data about all-cause mortality were collected at the end of the follow-up period. The mean follow-up period was 56 ± 22 months.

**Results:**

In this cohort, 58% of patients with acute ischemic stroke or TIA had heart dysfunction. Survival analysis showed that heart dysfunction did not predict all-cause mortality in this cohort. Furthermore, in multivariate regression analysis age (HR 5.401, Cl 1.97-14.78, p < 0.01), smoking (HR 3.181, Cl 1.36-7.47, p < 0.01), myocardial infarction (HR 2.826, Cl 1.17-6.83, p < 0.05) were independent predictors of all-cause mortality.

**Conclusion:**

In this population with acute ischemic stroke or TIA and without non-valvular atrial fibrillation, impaired heart function does not seem to be a significant predictor of all-cause mortality at long-term follow-up.

## Background

Ischemic stroke and heart failure are major causes of morbidity and mortality [1]. Previous studies have suggested that heart failure is associated with increased risk of ischemic stroke [2-7]. Risk factors for heart dysfunction are also hypertension, coronary artery diseases and diabetes mellitus. In a study of 1247 patients diagnosed with heart failure, Alberts et al. found that the risk of ischemic stroke was strongly increased shortly after the diagnosis and returned to normal within 6 months [3]. On the other hand, Witt et al. reported that the risk of suffering an ischemic stroke increased within the first 30 days after heart failure diagnosis and remained elevated during the 5-year follow-up period [6]. The somewhat different results of these two studies could be due to differences in heart failure populations. Even though atrial fibrillation is a well-recognized risk factor for both heart failure and ischemic stroke [8-11], it is still unclear if heart dysfunction *per se*, in the absence of atrial fibrillation, is associated with increased risk of ischemic stroke. Moreover, it remains unknown whether heart dysfunction *per se* may predict poor outcome in patients with acute ischemic stroke or transient ischemic attack (TIA).

Heart failure was associated with poor prognosis and dependency in ischemic stroke patients [12-15]. It is currently discussed whether there is an independent association between low left ventricular ejection fraction (LVEF) and mortality in patients with ischemic stroke. Such an association between LVEF and mortality has been reported in some studies but not in all [13,16].

The purpose of this study was to investigate whether heart dysfunction, as defined by echocardiographic findings, in the absence of atrial fibrillation, may predict long-term all-cause mortality in elderly patients with acute ischemic stroke or TIA.

## Methods

### Study cohort

We performed a retrospective survival analysis of patients admitted to the Stroke Unit, at Department of Medicine, Sahlgrenska University Hospital, in Gothenburg, from February 9, 2005 through May 31, 2009. Due to problems related to data management, no patients were included from January 1, 2007 until September 6, 2007.Patients with ischemic stroke or TIA were included if an echocardiography was performed during hospital stay. Patients with a medical history of atrial fibrillation, or who were found to have atrial fibrillation on the 24 hours cardiac monitoring after admission were excluded.

Stroke was diagnosed according to the World Health Organization definition of stroke [17]. TIA was defined as a neurologic deficit that resolved completely within 24 hours. Within 24 hours of admission, a cerebral computer-tomography (CT) scan was performed on all patients. Treatment with antiplatelet drugs was initiated if the CT scan excluded cerebral bleeding. On admission, stroke severity was assessed by using National Institute of Health Stroke Scale (NIHSS) [18,19]; functional status was estimated by using Modified Rankin Scale (mRS) [20,21] at 3-month follow-up.

Heart function was measured by echocardiography. Heart dysfunction was either categorized as systolic dysfunction or diastolic dysfunction. Systolic dysfunction was defined as LVEF <50% [22]. Diastolic dysfunction was defined as a combination of following criteria: 1) LVEF ≥50%; 2) Normal-sized left ventricle [LVDD ≤5.5 (cm)]; and 3) one of the following criteria: left ventricular hypertrophy; E/A ratio <0.8 or ≥1.5; S/D ratio <1 or >1; left atrium ≥34 (cm^2^) [23]. Echocardiography was performed by an echocardiography specialist who was blinded to the other variables examined in the study and, moreover, was independent of study team. Inter-observers were LVEF estimation/Simpson 22/27%, diastolic/systolic volume 14/22%, 2D analyzing (mitral ring) 13%, VTI LVOT 8%. Doppler echocardiography was used to obtain transmitral flow and pulmonary venous flow determined from the apical four chamber view. To record transmitral flow, the sample volume was carefully positioned at the tip of the leaflets of mitral valve. The following variables were evaluated as parameters of left ventricular filling: peak of early diastolic (E) and late diastolic (A) flow velocity (E/A ratio), and peak pulmonary venous flow velocity during ventricular systole (S), peak pulmonary venous flow velocity during ventricular diastole (D) (S/D ratio). All images were stored in the hospital central image database for later review. The final diagnosis was validated by a cardiologist. The study was approved by the Ethics Committee at the University of Gothenburg. The study protocol conformed to the ethical guidelines of the 1975 Declaration of Helsinki.

### Laboratory analyses at baseline

Serum-total cholesterol, hemoglobin, C-reactive protein (CRP), serum creatinine (S-creatinine), glomerular filtration rate (GFR), and cardiac troponin T (cTNT) were measured by routine laboratory methods within 24 hours of hospital admittance.

### Outcome data

Information on all-cause mortality was collected from medical records and the Swedish populations register at the end of the follow-up period.

### Statistics

The results are presented as percentage/mean **±** standard deviation (SD). Student’s unpaired t-test and, for discrete variables, the chi-square test, were used to assess statistical significance. Kaplan-Meier curves and Cox proportional-hazard regression model were used for survival analysis. The hazard ratios (HR) with confidence intervals (CI) and p-values are presented. *p* < 0.05 was regarded as statistically significant. The PASW Statistics 18 (SPSS Inc., Chicago, Illinois, USA) statistical package was used for all statistical analyses.

## Results

### Patient characteristics at baseline

Totally, n = 932 patients with acute ischemic stroke or TIA were admitted to the stroke unit. Among them, n = 132 patients with mean age 73 ± 9 were referred for echocardiography due to suspect onset of heart failure or due to suspect cardiac embolism, other than atrial fibrillation. N = 34 had documented atrial fibrillation and were thus excluded. Of the patients with heart dysfunction had 61 diastolic dysfunction and 16 systolic dysfunction.

Of the included patients with echocardiography, 58% were found to have heart dysfunction. Detailed clinical characteristics of the study population are presented in Table 1.

**Table 1:** Characteristics of the acute ischemic stroke or TIA cohort by heart dysfunction or normal heart function

	**Total**	**Heart dysfunction**	**Normal heart function**	**P-values**
	n = 132	n = 77	n = 55	
Age (mean ± SD)	73.3 ± 9.2	74.4 ± 8.0	71.7 ± 10.6	0.100
Male (%)	52.3	51.9	52.7	0.930
Stroke (%)	85.6	93.5^**^	74.5	0.002
TIA (%)	14.4	6.5^**^	25.5	0.002
NIHSS scores	3.4 ± 4.7	3.9 ± 5.1	2.9 ± 4.1	0.297
mRS at 3-months follow-up				
≤2 (%)	84.7	78.9^*^	92.7	0.030
>2 (%)	15.3	21.1^*^	7.3	0.030
All-cause mortality (%)	29.5	35.1	21.8	0.100
**Clinical examinations**				
Systolic BP (mmHg) (mean ± SD)	169.0 ± 29.5	170.0 ± 32.3	167.8 ± 25.4	0.680
Diastolic BP (mmHg) (mean ± SD)	94.2 ± 17.6	95.0 ± 19.6	93.1 ± 14.5	0.548
Heart rate (beats/min) (mean ± SD)	77.2 ± 18.0	77.8 ± 18.3	76.4 ± 17.8	0.660
**Laboratory variables**				
S-Creatinine (μmol/L) (mean ± SD)	84.1 ± 38.2	86.3 ± 43.8	81.0 ± 28.5	0.444
CRP (mg/L) (mean ± SD)	16.2 ± 32.4	16.7 ± 34.3	15.6 ± 29.7	0.859
cTNT (μg/L) (mean ± SD)	0.06 ± 0.29	0.08 ± 0.37	0.03 ± 0.04	0.417
**Echocardiogram parameters**				
LVEF (%)	57.6 ± 9.8	56.0 ± 12.0^*^	60.3 ± 3.2	0.017
LVDD (cm) (mean ± SD)	4.9 ± 0.8	4.9 ± 0.9	4.7 ± 0.7	0.438
Left atrium (cm^2^) (mean ± SD)	19.8 ± 4.4	20.7 ± 4.6^**^	18.3 ± 3.6	0.004
Right atrium (cm^2^) (mean ± SD)	16.2 ± 3.9	16.5 ± 4.2	15.7 ± 3.4	0.309
PA mmHg (mean ± SD)	31.0 ± 8.4	30.8 ± 6.9	31.4 ± 11.4	0.795
Stroke volume (mean ± SD)	72.7 ± 19.7	68.8 ± 18.5^*^	91.0 ± 15.7	0.037
E/A ratio (mean ± SD)	0.9 ± 0.4	0.9 ± 0.4	0.9 ± 0.3	0.988
S/D ratio (mean ± SD)	1.4 ± 0.5	1.4 ± 0.5	1.1 ± 0.1	0.324
**Cardiovascular risk factors**				
Smoking (%)	14.4	19.6	8.3	0.102
Hypertension (%)	59.1	59.7	58.2	0.858
Diabetes mellitus (%)	18.9	16.9	21.8	0.476
Hypercholesterolemia (%)^1^	13.9	15.5	11.8	0.558
**Cardiovascular diseases**				
Myocardial infarction (%)	15.9	19.5	10.9	0.184
Angina pectoris (%)	15.9	18.2	12.7	0.398
**Comorbidity**				
Pulmonary embolism (%)	1.5	0.0	3.6	0.092
Previous ischaemic stroke (%)	17.4	19.5	14.5	0.461
eGFR <60 (mL/min) (%)^2^	32.3	38.9	23.1	0.063
Anaemia (%)^3^	11.7	13.2	9.6	0.541
**Pre-stroke medications**				
ACE inhibitors (%)	19.7	22.1	16.4	0.416
Angiotensin receptor blockers (%)	18.2	14.3	23.6	0.170
Beta-blockers (%)	36.4	41.6	29.1	0.142
Diuretics (%)	26.5	28.6	23.6	0.527
Aspirin (%)	42.4	44.2	40.0	0.634
Warfarin (%)	0.8	1.3	0.0	0.396
Clopidogrel (%)	1.5	1.3	1.8	0.810
Aspirin + Dipyridamol (%)	1.5	1.3	1.8	0.810
Dipyridamol (%)	7.6	6.5	9.1	0.578
**Post-stroke medications**				
ACE inhibitors (%)	30.5	34.2	25.5	0.283
Angiotensin receptor blockers (%)	23.7	21.1	27.3	0.408
Beta-blockers (%)	44.3	51.3	34.5	0.056
Diuretics (%)	32.8	34.2	30.9	0.691
Aspirin (%)	82.4	81.6	83.6	0.760
Warfarin (%)	8.4	7.9	9.1	0.808
Clopidogrel (%)	3.1	5.3	0.0	0.084
Aspirin + Dipyridamol (%)	2.3	3.9	0.0	0.136
Dipyridamol (%)	46.6	39.5	56.4	0.056

NIHSS, National Institutes of Health Stroke Scale; mRS, modified rankin scale; BP, blood pressure; S-Creatinine, serum creatinine; CRP, C-reactive protein; cTNT, cardiac troponin T; LVEF, left ventricular ejection fraction; LVDD, left ventricular diastolic dimension; PA, pulmonary artery pressure; E/A ration, passive filling of the ventricle (early [E] wave) and active filling with atrial systole (atrial [A] wave); S/D ratio, systolic and diastolic component of pulmonary vein; eGFR, estimated glomerular filtration rate; ACE inhibitors; angiotensin-converting-enzyme inhibitors.

^1^Hypercholesterolemia defined as total serum-cholesterol >6 mmol/L.

^2^eGFR estimated by Cockcroft-Gault formula.

^3^Anaemia according to WHO definition, as hemoglobin <130 g/L (males) and hemoglobin <120 g/L (females).

^*^, *P* < 0.05 for comparison of heart dysfunction with normal heart function.

^**^, *P* < 0.01 for comparison between heart dysfunction with normal heart function.

### Survival data

The mean follow-up period was 56 ± 22 months (min 1, max 92). At the end of the follow-up period, 39/132 patients had died. The all-cause mortality was 35% among patients with heart dysfunction, and 22% among patients with normal heart function (n.s.). All-cause mortality was associated with higher age (p < 0.01), higher mRS at 3-month follow-up (p < 0.01), high CRP (p < 0.05), decreased LVEF (p < 0.05), smoking (p < 0.01), pre-stroke myocardial infarction (p < 0.05), pre-stroke angina pectoris (p < 0.05), eGFR <60 (mL/min) (p < 0.05), anaemia (p < 0.05), pre-stroke treatment with clopidogrel (p < 0.05) and no post-stroke treatment with aspirin (p < 0.05) (for details see Table 2).

**Table 2:** Characteristics of the acute ischemic stroke or TIA cohort by survival at the end of the follow-up period

	**Non-survivors**	**Survivors**	**P-values**
n = 132	n = 39	n = 93	
Age (mean ± SD)	77.5 ± 6.8^**^	71.5 ± 9.6	0.001
Stroke (%)	97.4^*^	80.6	0.012
TIA (%)	2.6^*^	19.4	0.012
mRS at 3-months follow-up			
≤2 (%)	69.2^**^	91.3	0.001
>2 (%)	30.8^**^	8.7	0.001
**Laboratory variables**			
CRP (mg/L)	28.0 ± 48.9^*^	11.4 ± 21.2	0.012
**Echocardiogram parameters**			
LVEF	55.0 ± 12.3^*^	58.8 ± 8.2	0.043
**Cardiovascular risk factors**			
Smoking (%)	29.6^**^	9.1	0.009
**Cardiovascular diseases**			
Myocardial infarction (%)	28.2^*^	10.8	0.012
Angina pectoris (%)	25.6^*^	11.8	0.048
**Comorbidity**			
eGFR <60 (mL/min) (%)^1^	47.2^*^	26.1	0.023
Anaemia (%)^2^	21.1^*^	7.8	0.033
**Pre-stroke medication**			
Clopidogrel (%)	5.1^*^	0.0	0.028
**Post-stroke medication**			
Aspirin (%)	71.1^*^	87.1	0.029

Only variables that are significant are listed in the table.

mRS, Modified rankin scale; CRP, C-reactive protein; LVEF, left ventricular ejection fraction; eGFR, estimated glomerular filtration rate.

^1^eGFR estimated by Cockcroft-Gault formula.

^2^Anaemia according to WHO definition, as hemoglobin <130 g/L (males) and hemoglobin <120 g/L (females).

^*^, *P* < 0.05; ^**^, *P* < 0.01 non-survivors compared with survivors.

### Predictors of mortality

In a univariate regression analysis of the whole ischemic stroke and TIA cohort (n = 132), the following factors were found to be significant indicators of poor survival: age, mRS >2 at 3-month follow-up, pre-stroke anaemia, CRP, smoking, pre-stroke myocardial infarction, and pre-stroke angina pectoris. Use of post-stroke aspirin was associated with better survival. However, when the variables which were significant in univariate analyses were further tested in a multivariate regression model, only age, smoking and pre-stroke myocardial infarction, were independently associated with all-cause mortality, while use of post-stroke aspirin was identified as a protective factor (Table 3). Heart dysfunction was not identified as an independent predictor of all-cause mortality.

**Table 3:** Univariate- and multivariate regression analyses of all-cause mortality in the total ischemic stroke or TIA cohort (n = 132)

**Univariate**	**HR**	**95% CI**	**P-values**
Age (years)	4.903	2.15-11.19	<0.000
mRS >2 at 3-months follow-up (%)	4.163	2.07-8.36	<0.000
Anaemia (%)	2.462	1.13-5.39	0.024
CRP (mg/L)	2.070	1.04-4.11	0.037
Smoking (%)	2.327	1.01-5.39	0.049
Myocardial infarction (%)	2.405	1.20-4.84	0.014
Angina pectoris (%)	2.530	1.23-5.22	0.012
Post-stroke Aspirin (%)	0.360	0.18-0.73	0.005
Heart dysfunction (%)			n.s
**Multivariate**	**HR**	**95% CI**	**P**
Age (years)	5.401	1.97-14.78	0.001
mRS >2 at 3-months follow-up (%)			n.s.
Anaemia (%)			n.s.
CRP (mg/L)			n.s.
Smoking (%)	3.181	1.36-7.47	0.008
Myocardial infarction (%)	2.826	1.17-6.83	0.021
Angina pectoris (%)			n.s.
Post-stroke Aspirin (%)	0.312	0.13-0.77	0.011

mRS, Modified rankin scale; CRP, C-reactive protein.

In patients with heart dysfunction (n = 77), univariate regression analysis showed that age, NIHSS score, mRS >2 at 3-month follow-up and previous myocardial infarction were all significant prognostic indicators associated with poor survival. Use of post-stroke aspirin was associated with better survival. When the significant variables from this analysis were further tested in the multivariate regression analysis (Table 4) only mRS >2 at 3-month follow-up was found to be an independent predictor of all-cause mortality; use of post-stroke aspirin was identified as protective factor.

**Table 4:** Univariate- and multivariate regression analysis of all-cause mortality in the acute ischemic stroke or TIA cohort with heart dysfunction (n = 77)

**Univariate**	**HR**	**95% CI**	**P-values**
Age (years)	3.759	1.58-8.96	0.003
NIHSS score	1.123	1.03-1.23	0.012
mRS >2 at 3-months follow-up (%)	5.914	2.60-13.46	<0.000
Myocardial infarction (%)	2.641	1.14-6.10	0.023
Post-stroke Aspirin (%)	0.292	0.12-0.69	0.005
**Multivariate**	**HR**	**95% CI**	**P**
Age (years)			n.s.
NIHSS score			n.s.
mRS >2 at 3-months follow-up (%)	6.133	2.12-17.76	0.001
Myocardial infarction (%)			n.s.
Post-stroke Aspirin (%)	0.174	0.06-0.54	0.003

mRS, Modified rankin scale; NIHSS, National Institutes of Health Stroke Scale.

## Discussion

This study investigated the impact of impaired heart function on long-term all-cause mortality in a population with acute ischemic stroke or TIA. Our results show that heart dysfunction does not seem to be a predictor of all-cause mortality in this clinical setting.

Heart failure and ischemic stroke are not only two of the most common diseases in elderly people, but also two of the most common causes of death [1,4,19,24-26]. Some studies have suggested that a strong association would exist between heart failure and ischemic stroke [2-7]. For example, The Framingham study found heart failure to be one of the major risk factors for ischemic stroke [5]. Both CHADS_2_ score and CHADS_2_-Vasc score [27] take heart failure into account as one of the risk factors for stroke. However, these scores address heart failure patients with atrial fibrillation. Since heart failure often occurs in conjunction with atrial fibrillation, it is difficult to estimate the exact contribution of each of these factors to the risk of stroke and/or decreased survival after stroke. Moreover, heart dysfunction, cardiac remodeling and atrial fibrillation may all contribute to the risk of ischemic stroke and affect the prognosis thereafter.

As previously shown [8-10], the incidence of atrial fibrillation, which increases with age [11,28], is not only an independent risk factor for ischemic stroke, but also for worse outcome after ischemic stroke. In one of our previously published works, on data from the Swedish Heart Failure Registry, almost 50% of the patients with heart failure had associated atrial fibrillation [24]. Thus, it is difficult to study how heart dysfunction, beyond atrial fibrillation, affects prognosis after acute ischemic stroke or TIA.

At present, there are limited studies about impact the effect of heart dysfunction, in the absence of atrial fibrillation, on the long-term prognosis of ischemic stroke. A few previous studies suggested that neurologic deficits induced by stroke are more severe in patients with heart failure, regardless the type of heart dysfunction (systolic or diastolic) [29]. In the Northern Manhattan stroke study [7], evaluating 323 stroke patients from a mixed-ethnic region, heart failure was found to be a significant predictor of mortality.

Thus, the impact of heart dysfunction *per se*, in the absence of atrial fibrillation, on stroke and mortality is less clear. In the present work, we aimed to assess if heart dysfunction alone may affect the prognosis of ischemic stroke or TIA.

In some recent studies heart failure was associated with poor prognosis in ischemic stroke patients [12-16].

It has been suggested that patients with heart failure who have an incident ischemic stroke have higher risk for midterm dependency and early mortality due to index stroke severity. Heart failure seems even to be a predictor for long-term mortality [13-16]. Underlying cardiovascular risk factors such as hypertension, valvular disease and coronary artery disease may explain worse prognosis in patients with ischemic stroke and history of heart failure. Regardless of LVEF in ischemic stroke patients, have heart failure in some studies been found to be associated with more severity, recurrence risk and early- and long-term mortality [13].

Dependency has been reported to be associated with heart failure, stroke severity and age [14] and it has recently been demonstrated that heart failure, atrial fibrillation and prestroke dementia are risk factors for death within 1 month of a stroke event [15].

In ischemic stroke patients with low LVEF (≤35) it seems plausible that stroke severity, age and atrial fibrillation are independent predictors for 1-year mortality [16].

To achieve this purpose our study design was straightforward by excluding patients with concomitant atrial fibrillation. Our results indicate that heart dysfunction is not an independent predictor of all-cause mortality during long-term follow up in this cohort. Instead, we found that higher age, smoking, and pre-stroke myocardial infarction and none use of post-stroke aspirin [30-32] are independent predictors of mortality in patients with acute ischemic stroke or TIA, without atrial fibrillation. Lip et al. [33] found that previous stroke/TIA was a predictor of mortality and of the composite end-point of stroke together with mortality in heart failure patients without atrial fibrillation, especially during the first 30 days following initial diagnosis of heart failure. The difference between our study and the study of Lip et al. is that our follow-up time is longer. There are no data in the latter study on echocardiography parameters, so LVEF cannot be compared between the two studies.

It is worthwhile to point out that in our study only 7% of the total acute ischemic stroke or TIA cohort had LVEF ≤30%. This might be due to the fact that patients with severe heart dysfunction often were associated atrial fibrillation and therefore were excluded. Moreover, patients with already known heart failure were not regularly referred to echocardiography during hospital stay in daily practice.

It may be hypothesized, that the lack of association between mortality and heart dysfunction/heart failure found in the present study may be explained by less severe strokes in the present population. Also many patients were chronically treated with long-term statins and antihypertensives, which may have had a beneficial effect on outcome. Another conceivable mechanism may be that non-valvular atrial fibrillation *per se* is a stronger risk factor for mortality and dependency than heart failure. Since heart failure patients with non-valvular atrial fibrillation were included in previous studies, but not in the present one, our inconsistent results may at least partly be explained by differences in study population.

Despite the limited sample size, our study is of potential interest since it examines the relationship between heart dysfunction without concomitant non-valvular atrial fibrillation – assessed immediately after onset of ischemic stroke or TIA – and long-term stroke prognosis. Moreover, our study is to our knowledge among the first studies to assess impact of heart dysfunction without concomitant atrial fibrillation on prognosis of patients with acute ischemic stroke or TIA.

### Limitations

Our sample size was relatively small, since we excluded all patients with atrial fibrillation and all patients not examined with echocardiography during hospital stay. Moreover, a possible bias in the present study may be that patients with previously known heart failure were not regularly referred to echocardiography during hospital stay. Moreover, we did not control for atrial fibrillation or other comorbidities which might have debuted during the follow-up period and thus, may affect long-term prognosis. Finally, included patients did not have higher NIHSS points i.e. less severe strokes.

## Conclusion

In conclusion, heart dysfunction, in the absence of atrial fibrillation, does not seem to predict long-term all-cause mortality in this population with acute ischemic stroke or TIA. However, due to our small sample size any conclusion must be drawn with great caution. A larger, prospective controlled study is warranted in order to confirm our findings.

## Abbreviations

ACE inhibitors: Angiotensin-converting-enzyme inhibitors; BP: Blood pressure; CI: Confidence intervals; CRP: C-reactive protein; CT: Cerebral computer-tomography; cTNT: Cardiac troponin T; E/A ratio: Peak of early diastolic (E) and late diastolic (A) flow velocity; eGFR: Glomerular filtration rate; HR: Hazard ratios; LVDD: Left ventricular diastolic dimension; LVEF: Left ventricular ejection fraction; mRS: Modified Rankin Scale; NIHSS: National Institute of Health Stroke Scale; PA: Pulmonary artery pressure; S-creatinine: Serum creatinine; S/D ratio: Peak pulmonary venous flow velocity during ventricular systole (S), peak pulmonary venous flow velocity during ventricular diastole (D); SD: Standard deviation; TIA: Transient ischemic attack.

## Competing interests

There is no relationship with the industry and no conflict of interest.

## Authors’ contributions

AH has done acquisition of echocardiography data, analysis and interpretation of the data, statistical analysis, and writing, drafting, and critical revising of the manuscript. MLXF has worked with the conception and design of the research, drafting and critical revising of the manuscript. CH has provided the epidemiological data and has worked with English language corrections, drafting and revising of the manuscript. LB has provided epidemiological data, and has worked with drafting and critical revision of the manuscript. BA has provided the epidemiological data, has worked with conception and design of the study, and with drafting and critical revising of the manuscript. All authors have read and approved the final manuscript.

## Pre-publication history

The pre-publication history for this paper can be accessed here:

http://www.biomedcentral.com/1471-2377/13/122/prepub
